# Measuring the effect of nurse practitioner (NP)-led care on health-related quality of life in adult patients with atrial fibrillation: study protocol for a randomized controlled trial

**DOI:** 10.1186/s13063-017-2111-4

**Published:** 2017-08-03

**Authors:** Marcie J. Smigorowsky, Colleen M. Norris, Micheal Sean McMurtry, Ross T. Tsuyuki

**Affiliations:** 1 Mazankowski Alberta Heart Institute, 2C2, WMC, 8440 – 112 Street, Edmonton, AB T6G 2B7 Canada; 2 Faculty of Nursing, University of Alberta, 4-127, Clinical Sciences Building, Edmonton, AB T6G 2G3 Canada; 3 Division of Cardiology, University of Alberta, 2C2, WMC, 8440 – 112 Street, Edmonton, AB T6G 2B7 Canada; 4 Faculty of Medicine and Dentistry, EPICORE Centre, University of Alberta, 362 Heritage Medical Research Centre, Edmonton, AB T6G 2S2 Canada

**Keywords:** Atrial fibrillation, Health-related quality of life, Nurse practitioner, Sustainable models of healthcare, Patient satisfaction

## Abstract

**Background:**

Atrial fibrillation (AF) is a common arrhythmia associated with significant morbidity, mortality, and healthcare resource use. The prevalence of AF is increasing with a growing and aging population, and timely access to care for these patients is a concern. Nontraditional models of care delivery, such as nurse practitioner (NP)-led clinics, may improve access to care and quality of care, but they require formal assessment. The objective of this study is to assess the effect of NP-led care on the health-related quality of life (HRQoL) of adult patients with AF.

**Methods/design:**

We plan a randomized controlled trial comparing NP-led care vs. standard care. Inclusion criteria are ≥18 years of age, documented nonvalvular AF, willingness to give informed consent, and capacity to complete questionnaires. Patients referred for electrophysiological intervention who are clinically unstable or unable to attend follow-up visits will not be eligible to participate. Patients will be asked for verbal consent during the initial triage phone call from the nurse. Randomization will occur via a secure website. The intervention includes an NP consult, including medical history, physical examination, patient teaching, treatment plan, and follow-up at 3 and 6 months. The control arm involves usual cardiologist consultation with follow-up determined by the cardiologist’s practice pattern. The primary outcome will be the difference in change in Atrial Fibrillation Effect on Quality of Life Survey scores at 6 months between groups. Secondary outcomes will include difference in change of EQ-5D scores at 6 months between groups, difference in composite outcomes of death resulting from cardiovascular cause, hospitalizations and emergency department visits between groups, and satisfaction with NP-led care measured by the Consultant Satisfaction Questionnaire. A sample size of 70 per group will ensure adequate power despite a potential 10% loss to follow-up.

**Discussion:**

Our study will determine the effect of NP-led AF care on HRQoL in patients with AF, as well as measure its impact on relevant outcomes such as death, hospitalization, and emergency department visits. Our findings may have implications for delivery of care to patients with AF.

**Trial registration:**

ClincalTrials.gov, NCT02745236. Registered on 16 April 2016.

**Electronic supplementary material:**

The online version of this article (doi:10.1186/s13063-017-2111-4) contains supplementary material, which is available to authorized users.

## Background

Atrial fibrillation (AF) is the most common arrhythmia, and it is increasing in prevalence in a growing and aging population [1]. Currently in Canada, there are approximately 350,000 people living with AF [2], but this number is expected to rise. Many countries are experiencing a healthcare crisis, including Canada [3–5]. Increasing healthcare demands in an already overwhelmed system require new methods of care delivery to be examined.

AF is a chronic disease associated with devastating complications such as stroke and heart failure [6]. Patients with AF have a three to five times greater risk of stroke, with strokes typically larger and associated with higher mortality than in patients without AF [1]. There are evidence-based guidelines for treatment for AF in Canada [6] that promote evidence-based practice and improved patient outcomes, including improved quality of life (QoL) and symptom control. Early intervention and individualized assessment are fundamental for optimal AF management.

Currently, healthcare in Canada comprises more than 40% of all government funding [7], and these costs are judged to be unsustainable [8]. Estimates indicate AF hospitalizations cost the Canadian healthcare system $815 million, with most costs driven by poorly managed AF [9]. In addition, projections suggest that there will be a dramatic inability to meet the future demand for healthcare traditionally provided by a physician [10]. These fiscal and demographic realities support the need for evaluation of new models of care.

Nurse practitioners (NPs) are highly trained clinicians and independent healthcare professionals who work in collaboration with other members of the healthcare team to manage a patient’s full spectrum of healthcare needs [11]. In Alberta, “NP” is a protected title whose scope of practice is legislated pursuant to the Health Professions Act. NPs provide comprehensive health assessment, as well as diagnose, treat, and manage disease, within a holistic model of care [11]. Each NP is accountable for determining his or her own expertise level in specific competencies and when it is appropriate to be involved or refer patients to other healthcare providers [12].

Patients are often more satisfied with NP-led care than with physician-led care [13, 14]. This may be related to NPs’ spending more time engaging with patients in individualized treatment options through patient education and counseling [15]. NP patient-centered care may improve adherence to treatment plans [16–18]. NP care also may improve clinical and patient-reported outcomes as well as lead to substantive cost savings [19–30]. However, despite potential advantages, there are often barriers to broad adoption of NP-led care in many environments [31].

To the best of our knowledge, there has been one only randomized controlled trial (RCT) to date involving assessment of nurse-led care vs. standard physician care for patients with AF. Researchers at a Dutch outpatient hospital clinic randomized 714 patients with AF into 2 equal groups for 2 years [32]. The control arm included a 20-minute initial cardiologist consult and 10-minute follow-up appointment as required. The intervention arm comprised an initial consultation in the nurse-led clinic with diagnostic testing completed prior to the visit. Treatment was guided by AF-specific decision support software. At the end of the consult, a cardiologist would review the patient and care plan. Follow-up visits were held at 3, 6, and 12 months and then every 6 months thereafter. The primary endpoint, a composite of cardiovascular hospitalization and cardiovascular death, occurred in 14.3% in the nurse-led care group compared with 20.8% in the usual care group (HR 0.65, 95% CI 0.45–0.93, *p* = 0.017). Cardiovascular death occurred in 1.1% of the nurse-led care group vs. 3.9% in the usual care group (HR 0.28, 95% CI 0.09–0.85, *p* = 0.025). Cardiovascular hospitalization occurred in the 13.5% in the nurse-led care group vs. 19.1% in the usual care group (HR 0.66, 95% CI 0.46–0.96, *p* = 0.029). Adherence to clinical guidelines was significantly higher in the nurse-led care group. The researchers concluded that nurse-led care for patients with AF was superior to usual care provided by a cardiologist in this setting. Some important limitations of the study were that complex patients were excluded from participating, and a cardiologist was still required to review the patient’s care. Important remaining questions include whether other outcomes are improved, such as health-related quality of life (HRQoL), the patient’s perception of the quality of care received, and whether a more independent practitioner such as an NP would achieve similar results in patients. The objective of this study is to assess the effect of NP-led care on the HRQoL of adult patients with AF.

## Methods/design

We hypothesize ambulatory patients with AF whose care is managed by an NP will have improved HRQoL as measured by Atrial Fibrillation Effect on QualiTy of life Survey (AFEQT) scores compared with patients receiving standard care.

### Design and setting

**Fig. 1:**
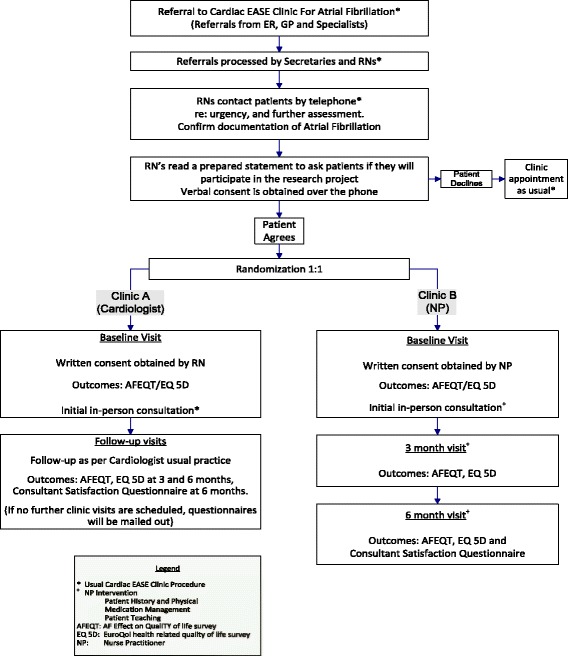
Patient flow diagram. AFEQT Atrial Fibrillation Effect on QualiTy of life survey, EASE Ensuring Accessed and Speedy Evaluation, ER Emergency room, GP General practitioner, NP Nurse practitioner, RN Registered nurse

We propose a prospective RCT with two equal groups testing for superiority (*see* Fig. 1). The RCT will be conducted in the Cardiac Ensuring Accessed and Speedy Evaluation (EASE) Clinic, a multidisciplinary general cardiology outpatient referral clinic in a large tertiary care hospital in Alberta [33]. The clinic’s normal practice is for patients to be triaged by registered nurses (RNs) who follow algorithms based on American Heart Association and Canadian Cardiovascular Society (CCS) guidelines. Diagnostic testing is completed prior to the initial clinic visit to decrease time to follow-up. Patients are assessed in the clinic either (1) by an RN or a doctor of pharmacy and a cardiologist or (2) solely by an NP. Follow-up, if required, is either in the cardiologist’s own clinic or the NP clinic. Patients who do not require follow-up will be returned to their family physician’s care. The hospital uses an electronic medical record that incorporates scheduling of diagnostic testing, clinic appointments, and communications (letters, patient’s health history). In the context of this study proposal, *standard care* refers to regular clinic processes as described above.

### Study population

All adult patients who are referred for assessment for AF to the EASE clinic will be asked to participate in the study. Inclusion criteria are patients aged 18 years or older, with documented AF, able to provide informed consent, and able and willing to complete the study questionnaires on their own or with assistance.

Exclusion criteria are patients referred for atrioventricular node ablation or pulmonary vein isolation, patients who have failed rate control or antiarrhythmic medications, or patients who have moderate to severe mitral or aortic valvular heart disease. Patients with unstable AF or who cannot or are unwilling to attend follow-up appointments are also excluded. Study criteria will be reviewed with Cardiac EASE RNs on an ongoing basis to assist with study recruitment.

After verbal consent is obtained during the telephone triage call, the RN will randomize patients on a secure website. Block randomization (using variable block sizes) will be used to ensure there are equal participants in the intervention and control groups and to further conceal allocation. The patient will be scheduled in the determined clinic within 4–6 weeks from the date of referral, consistent with CCS guidelines.

Prior to the initial clinic visit, written consent will be obtained by a research assistant along with the baseline questionnaires. Questionnaires will also be completed at 3- and 6-month in-person follow-up appointments. Patients who are not being seen in follow-up will have the questionnaire mailed to them. Patients will receive telephone reminders in 2 and 4 weeks if the questionnaires have not been returned.

### Intervention

The intervention group will receive NP-led care. The initial visit is with an experienced NP with extra training in AF management. A complete baseline history and physical examination will be completed to determine a plan of care based on current CCS AF guidelines. CHADS_2_/CHA_2_DS_2_-VASc score [34] will be calculated to identify the risk of stroke for each patient. To assist with determining risk for increased potential for bleeding, the HAS-BLED score [35] will be calculated. Canadian Cardiovascular Society Severity in Atrial Fibrillation Scale scores [36] will also be calculated to identify symptom severity of AF. A cardiologist will be consulted if the patient develops heart failure; medication intolerances (which limit medical management) requiring assessment for treatment with amiodarone, electrical cardioversion, or pulmonary vein isolation; or other serious complications. The NP will also provide individualized patient education (“What is AF?,” “AF management and complications”). Patients will be given a written treatment plan at the end of the consult to assist with patient compliance, self-management, and knowledge retention. Patients will also be provided with clinic contact information for future needs.

The NP will see the patient in follow-up at 3 and 6 months; however, if the patient’s condition requires closer follow-up, timing will be adjusted and documented. The patient’s history will be reviewed to determine if the patient has been hospitalized or has had any major adverse cardiovascular events. A provincial electronic medical record will also be reviewed for prescribed medications, dates of hospitalizations and emergency room visits, laboratory blood work results (e.g., international normalized ratio, troponin, brain natriuretic peptide, hemoglobin), and diagnostic tests (echocardiograms, chest x-rays, medical consultations, computed tomographic scans, and 12-lead electrocardiogram tracings).

**Fig. 2:**
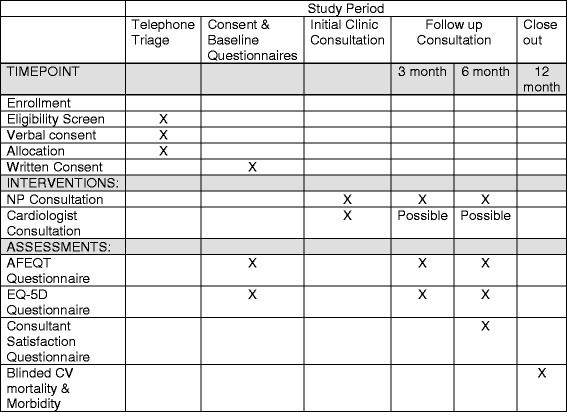
Standard Protocol Items: Recommendations for Interventional Trials (SPIRIT) diagram. AFEQT Atrial Fibrillation Effect on QualiTy of life survey, CV Cardiovascular

The control group will receive standard care by a cardiologist in the EASE Clinic. The cardiologist will determine AF management and follow-up requirements as per their usual practice. The patient’s care will be referred back to the family physician if no follow-up is required. The schedule of enrolment, intervention, and assessments of the complete study protocol (according to Additional file 1: Standard Protocol Items: Recommendations for Interventional Trials [SPIRIT] checklist) is shown in Fig. 2.

### Outcomes

The primary outcome is the difference in change in AFEQT scores from baseline to 3 months and 6 months between the intervention and control groups. AFEQT is an AF-specific questionnaire [37] for use with patients with any type of AF. AFEQT is a simple survey with 20 questions based on a 7-point Likert scale covering 3 domains. The questionnaire should take about 5 minutes to complete. Four questions assess AF-related symptoms, eight questions evaluate daily functioning, and six questions evaluate AF treatment concerns. Two questions assessing satisfaction with treatment are not included in the overall score. Questions 1–18 are included in the overall scoring of the questionnaire. A score of zero corresponds to complete disability, whereas a score of 100 corresponds to no disability. The AFEQT has been shown to have good reliability, test-retest reliability, construct validity, and responsiveness as well as discriminatory properties between clinically different groups [37]. The lowest global score is associated with the patients with severe symptoms related to AF. The AFEQT has also been shown to be responsive to change [37].

### Secondary outcomes include

1. *Difference in change in EQ-5D from baseline to 6 months between intervention and control groups*: The EQ-5D is a simple five-question general QoL questionnaire. It assesses five dimensions of QoL: mobility, self-care, usual activities, pain/discomfort, and anxiety/depression. It also has a visual analogue scale on which patients self-rate their overall health. A utility of 1 represents full health, whereas a utility of zero represents a state equivalent to death [38].
2. *Difference in composite outcomes of death from cardiovascular causes, cardiovascular hospitalization, and emergency room visits between the intervention and control groups (for ischemic stroke, heart failure, acute myocardial infarction, systemic embolism, major bleeding, severe arrhythmic events, and life-threatening adverse effects of drugs)*: MSM will chair a blinded committee to determine this outcome.
3. *Satisfaction with healthcare provider care will be assessed as measured by the overall mean score of the Consultation Satisfaction Questionnaire (CSQ) completed at the 6-month follow-up visit*: The CSQ is a self-administered tool with 18 questions using a 5-point Likert scale ranging from strongly agree to strongly disagree [39]. Three factors are considered: (a) professional aspects of the consultation, (b) depth of patient relationship, and (c) perceived length of consultation. There are three questions related to overall general satisfaction. Higher scores indicate higher satisfaction.

### Sample size

A minimally important difference (MID) for change in AFEQT score was not identified in a review of the literature. In the initial validation study [37], the SD for change from baseline to 3 months in the medically managed group was 20.0 (overall AFEQT global score). This group is most like the projected population for this study, and that value was therefore used as the SD for sample size calculations. We consulted Dr. Paul Dorian, an experienced arrhythmia specialist, AF QoL researcher, and coauthor of the AFEQT questionnaire, who suggested that the MID for AFEQT is 12. As such, we chose an effect size of 12 (i.e., NP-led care would improve AFEQT scores by at least 12). A sample size of 64 participants in each group will detect an MID of 12 in AFEQT scores (SD 20, two-tailed *t* test, 80% power, and significance level of 5%). Seventy patients per group (total 140) will be recruited to allow for 10% loss to follow-up.

All sociodemographic and clinical characteristics for the intervention (NP-led care) and control groups at baseline will be summarized using mean ± SD for continuous variables and the observed counts and percentages for categorical variables. For the main outcome, analysis of covariance (ANCOVA) will be used to assess change in AFEQT scores over time: baseline to 3 months and 3 months to 6 months (repeated dependent measures) in both the intervention and control groups. ANCOVA assumes normal distribution of the data as well as homogeneity of variance and the groups being balanced. Adjustment for pretest scores will identify whether the postintervention difference in scores is truly a result of the intervention. ANCOVA will also account for variation around the posttest means that comes from the variation attributed to the patients AFEQT scores started at baseline. Previously, it has been shown with other HRQoL scores that data can be skewed because of ceiling or floor effects resulting from extreme values and unequal distances between values on ordinal scales [40]. However, the central limit theorem states that the means will be normally distributed regardless of the original distribution when there are at least 30 per group [41]. This study is projected to have 2 equal groups with 70 participants. If the initial data analysis reveals nonnormal distributions and skewed data, further analysis will be completed to determine if transformation of the data is required or if other statistical analysis would be more appropriate. An independent *t* test will be used to assess the difference in means between NP care and physician-led care. Kaplan-Meier survival analysis will be used to evaluate the composite endpoint of cardiovascular death and hospitalizations determined by blinded assessors. Multivariate analysis will be used to adjust for possible differences in baseline characteristics and scores for any significant variables. Consultant satisfaction will be determined by comparing the means with independent *t* tests. A *p* value less than 0.05 will be considered statistically significant. All analyses will be based on the intention-to-treat principle.

Missing data will be replaced with the overall mean of the missing variable. This technique is known to accurately identify the mean but will underestimate the SD, making the CI overly optimistic [42]; however, other options also have imperfections. This will need to be considered in the final analysis. All analyses will be performed with the latest version of IBM SPSS statistical software (IBM, Armonk, NY, USA).

Data entry will be completed in a mature, secure web application specifically developed for surveys and databases. An application has been specifically designed for this study with limits built into data entry fields to limit errors. Data entry will be completed by MJS with random data entry checks done by a specific Epidemiology Coordinating and Research (EPICORE) Centre staff member. The final dataset will be available only to MJS and a specific EPICORE Centre staff member. We did not feel that a data monitoring committee was needed for the following reasons. First, the NP intervention is already part of how care is delivered at our institution. We are simply evaluating it. Second, because this is a trial of a treatment approach, we did not feel that data monitoring would be necessary (and our research ethics board did not require it).

### Ethical considerations

This research protocol has been approved by the health research ethics board at the University of Alberta. The patients will be required to read and understand the clinical trial information sheet and provide consent to participate. All patient information and study questionnaires will be treated with confidentiality and locked in a secure data storage facility at the EPICORE Centre (www.epicore.ualberta.ca). All information will be de-identified to maintain confidentiality. Modifications to any part of the study protocol will be resubmitted to the health research ethics board at the University of Alberta. Participants will be notified if applicable.

## Discussion

Currently, there is little evidence identifying a model of care that is sustainable and improves the QoL of patients with AF. There are several known benefits of NP-led care for other chronic disease states [23–25], and it is therefore reasonable to assume similar benefits could be attained with NP-led care for patients with AF. NPs are independent practitioners working collaboratively with physicians, and therefore the potential exists to decrease wait times for patients to be seen. This is extremely important for stroke risk assessment, to reevaluate patients with increasing symptoms to adjust medication regimes, or to arrange for interventions such as cardioversion or cardiac electrophysiology interventions. Both interventions have the potential to make a strong impact on patient outcomes as well as on the healthcare system, limiting emergency room visits and hospital admissions. Earlier appropriate management should also produce fewer devastating, costly complications of stroke and heart failure. Some Canadian centers already use RNs or NPs in their clinic, but without complete evidence of their effectiveness. Our proposed trial will help by evaluating a framework of care and determining its impact on HRQoL.

We have chosen to conduct a superiority RCT design instead of a noninferiority design because we feel that NP care may offer some important patient-focused advantages over usual physician-based care. The primary outcome is a patient-important outcome rather than a clinical outcome. (Had we wished to evaluate clinical outcomes, we agree that it would have been reasonable to design a noninferiority trial of NP-led care vs. standard care.) NP-led care, as alluded to earlier, has consistently been shown to be rated higher in patient satisfaction. This may in part be due to the holistic model of care followed by NPs. It engages patients in their healthcare plans and addresses other psychosocial areas that may have an impact on their health. Ultimately, this may have an impact on their HRQoL, the primary outcome for this study.

The control group will receive usual care. In our case, patients are seen by a general cardiologist, and the details of the intervention and follow-up are left to the individual cardiologist. We make no attempt to protocolize this; it is truly usual care, and we feel that it is generalizable to the Canadian setting.

This research study is supported by the Cardiovascular Heath and Stroke Strategic Clinical Network, which is a network of professionals working toward better quality and outcomes for cardiovascular health in Alberta. Results will be presented at conferences and published in journals appropriate for research on AF and on NP roles. Plans will also be made to share findings with Alberta healthcare leaders and staff to support change in clinical practice if NP-led care is shown to be beneficial.

### Trial status

Study enrollment began August 2016 with 31 patients enrolled to date. Enrollment was limitied for a period of time due to staffing issues within the Cardiac EASE Clinic. These issues have been corrected and enrollment is progressing. We project enrollment will be completed by Spring 2018.

## Additional file

Additional file 1: SPIRIT checklist. (DOC 123 kb)

## Abbreviations

AF: Atrial fibrillation
AFEQT: Atrial Fibrillation Effect on QualiTy of life Survey
ANCOVA: Analysis of covariance
CCS: Canadian Cardiovascular Society
CHADS_2_: Congestive heart failure, hypertension, age ≥75 years, diabetes mellitus, prior stroke/transient ischemic attack
CHADSvasc: Congestive heart failure, hypertension, age ≥75 years, diabetes mellitus, stroke/transient ischemic attack, vascular disease (previous myocardial infarction, peripheral artery disease, or aortic plaque, age–65–74 years, female sex
CSQ: Consultation Satisfaction Questionnaire
EASE: Ensuring Accessed and Speedy Evaluation
CV: Cardiovascular
EPICORE: Epidemiology Coordinating and Research Centre
ER: Emergency room
GP: General practitioner
HAS-BLED score: Hypertension, Abnormal renal and liver function, stroke, bleeding, labile international normalized ratio, elderly, drugs or alcohol
HRQoL: Health-related quality of life
MID: Minimally important difference
NP: Nurse practitioner
QoL: Quality of life
RCT: Randomized controlled trial
RN: Registered nurse
SPIRIT: Standard Protocol Items: Recommendations for Interventional Trials
